# The Medico-Psychological Association and Its Nurses

**Published:** 1897-01-16

**Authors:** 


### Editor's Letter-Box.

[Our Correspondents are reminded that prolixity is a great bar to publication, and that brevity of style and conciseness of statement greatly facilitate early insertion.]

### THE MEDICO-PSYCHOLOGICAL ASSOCIATION AND ITS NURSES.

T. Outterson Wood, M.D., Member of the Council of the Medico-Psychological Association, writes: I regret to find in your issue of January 2nd an article on the above subject, evidently founded upon information respecting the Medico-Psychological Association, which is out of date. Especially is this so with regard to the nature and scope of the examination for the "Certificate for Proficiency in Nursing," given by the association, and it is upon this point alone I desire to say a few words to prevent misconception. It is stated that the knowledge required to pass the examinations can only be obtained by "cramming from a text-book." This is absolutely incorrect. The examinations do not consist of merely answering written questions, but it is necessary that the candidates shall in addition pass a *vivâ voce* examination, and give evidence of an intimate knowledge of practical nursing in its various branches by examination at the bedside of the patient. These examinations are of the highest grade, and compare favourably with any examinations of a similar nature. This you will see borne out by the paper of one of the recent examinations, which I now enclose. There are other statements which, in conjunction with the two concluding paragraphs, show such a regrettable want of knowledge with regard to the Medico-Psychological Association that I must pass them by without comment. I must ask you in fairness to publish this letter.

*** Dr. Wood does not touch the point at issue. No woman can be called a trained nurse who has not had practical experience in the work of the sick-room. Occasional demonstrations cannot take the place of this. Some of the Medico-Psychological nurses have had this training—others have not. The regulations do not insist on it. Our suggestion that asylum officers should discuss matters medical with their fellow practitioners in hospital and general practice was made in the interests of that medical spirit, the keeping up of which in asylums has, we believe, been a matter of consideration for the association.—Ed. *T. H.*

